# Plantar Myrmecia Wart: A Rare Case Report With Clinicopathologic Correlation and Literature Review

**DOI:** 10.7759/cureus.113839

**Published:** 2026-08-03

**Authors:** Tarang Patel, Gyanendra Singh, Yash Thesiya, Yashdeep Singh Pathania

**Affiliations:** 1 Pathology, All India Institute of Medical Sciences, Rajkot, Rajkot, IND; 2 Dermatology, Venereology and Leprology, All India Institute of Medical Sciences, Rajkot, Rajkot, IND

**Keywords:** histopathology, hpv-1, inclusion bodies, myrmecia wart, palmoplantar wart, wart

## Abstract

Myrmecia warts are a clinicopathological variant of deep palmoplantar warts caused by human papillomavirus type 1 (HPV 1), most commonly presenting on the plantar and palmar surfaces. The histopathological hallmark of these lesions is hyperkeratosis, acanthosis, papillomatosis with characteristic large intracytoplasmic inclusion bodies. We report a rare case of plantar Myrmecia wart in a 47-year-old male presenting with a solitary, slow-growing, exophytic verrucous lesion over the plantar aspect of the right foot of four years' duration. Given its chronicity, verrucous morphology, and lack of pigmentation, amelanotic melanoma was considered an important differential, prompting punch biopsy for confirmation. Histopathological examination revealed inclusion bodies confirming its diagnosis as Myrmecia warts and excluding malignancy. The patient was managed with radiofrequency ablation along with intralesional vitamin D3 and oral zinc sulphate. This case, along with a review of the literature, aims to consolidate existing k/nowledge on this entity in a single reference for the reader.

## Introduction

Myrmecia warts represent a distinctive clinicopathologic variant of deep palmoplantar verrucae caused by human papillomavirus (HPV), most commonly HPV-1 [1-3]. They present as painful, hyperkeratotic nodules and show pathognomonic large eosinophilic intracytoplasmic inclusions on histopathology [1,2,4]. Although well-described in the literature, these inclusions remain an uncommon finding, occasionally identified incidentally even at typical plantar sites [1,4].

We present a case of plantar Myrmecia wart, highlighting its classic histopathologic features and its importance in the differential diagnosis of verrucous lesions on the sole.

## Case presentation

**Figure 1:**
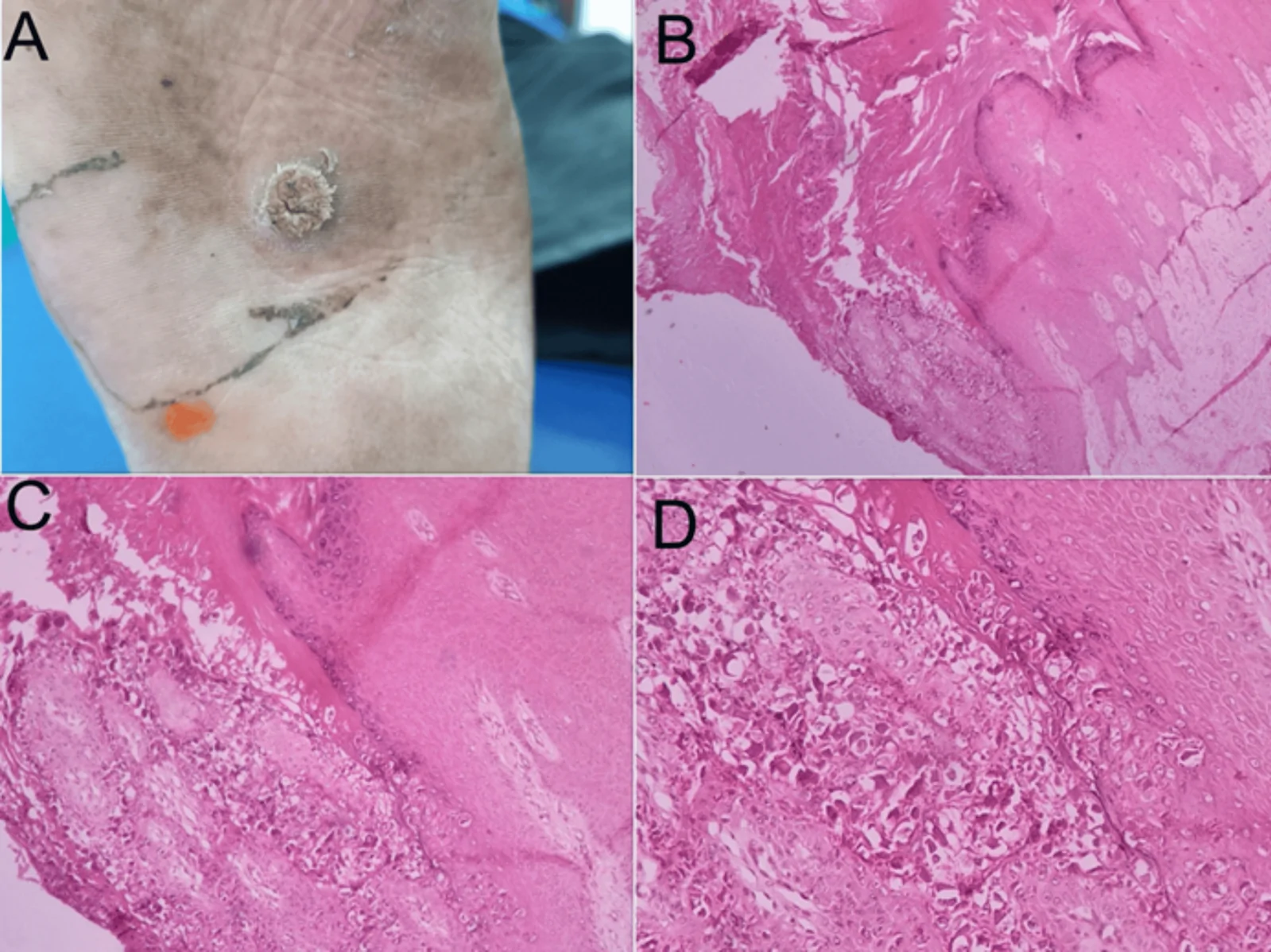
Clinical and microscopic images A: A well circumscribed, round exophytic papular lesion with rough to hard verrucous surface on the planter aspect of the foot; B: Section shows hyperplastic epidermis and dermis, with epidermis showing hyperkeratosis, papilomatosis and acanthosis. (Scanner, 4x, H&E); C: Section shows hyperplastic epidermis shows parakeratosis at places with inclusion bodies (10x, H&E); D: Section shows large eosinophilic intracytoplasmic inclusion bodies (40x, H&E)

A 47-year-old male presented to the dermatology department, AIIMS Rajkot, with a rough, hard growth over the plantar aspect of the right foot causing discomfort and pain on walking, of four years’ duration. Examination revealed a solitary, slow-growing exophytic papular lesion with a cystic, verrucous surface (Figure 1A), with no history of trauma or any similar lesion elsewhere. Given the chronicity, verrucous morphology, and lack of pigmentation, amelanotic melanoma was considered along with plantar wart, and punch biopsy was performed for definitive diagnosis. A 0.8 x 0.3 x 0.2 cm skin-covered greyish punch biopsy was received and processed for histopathology. Sections showed hyperplastic stratified squamous epithelium with hyperkeratosis, parakeratosis, papillomatosis, and acanthosis, along with multiple eosinophilic intracytoplasmic inclusion bodies and prominent keratohyaline granules (Figure 1B, 1C, 1D). The underlying dermis showed mild, non-specific lymphoplasmacytic infiltrate. No atypical cells, atypical melanocytes, or pigmentation were identified, excluding malignancy. A histopathological diagnosis of Myrmecia wart was made. HPV typing was not performed in the present case.

Following histopathological confirmation, the patient was started on radiofrequency (RF) ablation, with two sittings completed four weeks apart, along with intralesional vitamin D3 and oral zinc sulphate (50 mg twice daily) as immunotherapy. Follow-up was advised to assess treatment response and plan further RF sessions.

## Discussion

Deep palmoplantar warts (Myrmecia) arise from human papillomavirus belonging to the Papillomaviridae family, most commonly HPV-1 [1-3]. Deep palmoplantar warts are clinically and histologically distinct from the other variants of the warts. Unlike superficial, mosaic-type palmoplantar warts, deep palmoplantar warts are usually covered with a thick callus [5]. Our case presented as a rough, hardened papular growth rather than a discrete nodule.

Myrmecia warts most commonly occur on the palms and soles, as in our case. Unusual sites reported by other authors include the index finger [4], periungual [3], eyebrow, scalp, and forehead [1].

Histologically, these lesions show prominent hyperkeratosis, with eosinophilic cytoplasmic granules in the lower epidermis that enlarge and coalesce in the upper stratum malpighii to form large, irregularly shaped "inclusion bodies" [5]. Smaller intranuclear eosinophilic inclusions may also be seen, along with koilocytic change in places. Lyell et al. [6] termed these inclusion-containing warts as type I warts, reserving "type B" for cases lacking eosinophilic inclusions. They also distinguished type I inclusions from molluscum contagiosum bodies [6]. Tochiki et al. [7] noted typical HPV-1 cases show classic eosinophilic inclusions, whereas atypical cases with other HPV variants show pale-stained inclusions.

Devra et al. [4] discussed key histologic and clinical features between superficial mosaic-type palmar warts (verruca vulgaris), molluscum contagiosum, and deep palmoplantar warts. Based on the clinical and histopathological features summarized in Table 1, other common wart variants and amelanotic melanoma were excluded.

**Table 1:** Comparative clinicopathological features used to exclude differential diagnoses of Myrmecia wart.

	Verruca vulgaris	Deep palmoplantar warts	Verruca plana	Present case	Reference
Site	Dorsal fingers/hand	Palm and soles	Face, dorsa of hands	Plantar foot	[4,5]
Clinical features	Painless, circumscribed, firm, verrucous	Tender, occasionally red/swollen	Flat, smooth, slightly elevated	Painful minimal swelling	[5]
Number of the lesion	Single/multiple, coalescing	Mostly single	Multiple	Single	[5]
Histological features	Acanthosis, papillomatosis, hyperkeratosis, Foci of koilocytic cells, arborizing rete ridges	Acanthosis, papillomatosis, hyperkeratosis, abundant keratohyalin granules, large homogeneous inclusion bodies	Hyperkeratosis, acanthosis, no papillomatosis, diffuse vacuolization, basket-weave stratum corneum	Same as deep palmoplantar warts (see left)	[5]
HPV	2 (also 1,4,7,49)	1 (also 60,63,65)	3,10	Not done	[5]

Various reports of Myrmecia warts with review of the literature are summarized in Table 2. Following histopathological confirmation, the patient was started on both destructive and immunomodulatory therapy. RF treatment is the standard destructive therapy for plantar warts. Intralesional vitamin D3 was added to enhance local immune-mediated clearance, and oral zinc sulphate as a systemic immunomodulator.

**Table 2:** Published reports of Myrmecia wart.

No.	Authors & Year	Site/Location	No. of Cases	Key Findings/Notes
1	Wititsuwannakul et al. [1]	Eyebrow, scalp, forehead, leg	4	Incidental Myrmecia inclusions supporting latent HPV-1
2	Devra et al. [4]	Palmar (index finger)	1	Marked papillomatosis, hyperkeratosis, eosinophilic inclusions
3	Elkeeb et al. [2]	Plantar (left foot)	1	Classic plantar presentation, 25-year-old man
4	Holland et al. [3]	Periungual	Not specified	Tender nodules, deep palmoplantar type
5	Tochiki et al. [7]	Palmoplantar	Multiple (typical & atypical)	Typical vs. atypical inclusions, distinct HPV types
6	Egawa et al. [8]	Varies	Multiple (atypical)	Anatomical site as morphological factor
7	Yuki et al. [9]	Not specified	Not specified	Characteristic cytoplasmic inclusions
8	Suzuki et al. [10]	Palmoplantar	Multiple	Clinical, histologic, and IHC correlation
9	Lyell et al. [6]	Palmoplantar	Multiple	Foundational description of inclusion bodies (type I vs. B)

## Conclusions

Deep palmoplantar warts (Myrmecia warts) are benign, HPV-induced lesions that can present diagnostic challenges when they appear as chronic, exophytic, verrucous growths. Due to its chronicity and lack of pigmentation, amelanotic melanoma was considered a distant differential; hence, histopathology remains essential not only for excluding amelanotic melanoma, but also to confirm the diagnosis. The characteristic eosinophilic intracytoplasmic inclusion bodies are an important histopathological feature for Myrmecia warts. This case report summarizes much of the previously published literature on this entity while presenting a classic case of Myrmecia wart, adding to the growing body of literature on this uncommon condition.
